# The Modern Treatment of Wounds

**Published:** 1900-03

**Authors:** 


THE MODERN TREATMENT OF WOUNDS. By JOHN E. SUMMERS, JR., M. D., Surgeon-in-Chief to the Clarkson Medical Hospital; Attending Surgeon Douglas County Hospital; formerly Professor of the Principles and Practice of Surgery and Clinical Surgery, Omaha Medical College; ex-President of the Western Surgical and Gynecological Association, the Nebraska State Medical Society and the Omaha Medical Society. Medical Publishing Co, Omaha, 1899 Copyrighted, 1899, by J. E. Summers, Jr., M. D. Press of State Journal, Lincoln, Neb.

This volume came hustling in out of the west, unheralded, unknown and unlooked for. It came as a shock. It was a shock because all told, between the prettily-tinted green cloth covers, there are 149 pages, and that includes everything from soup to nuts; everything from preface to index; from the first bright word of joyous greeting to the last sobbing-note of a sad good-bye. For my part, I was generously inclined at first, for here, I thought, is a man who has condensed his work and put the modern treatment of wounds into small space. I skimmed through the volume. That was all I could stand; and of a truth was hardly necessary to skim it even once to get the cream. I expected a scintillating gem and I got one of those end-man diamonds which one sees in minstrel shows and vaudeville acts. Bill Nye used to tell a story of a funeral he once attended. It seems that a gentleman, by name McGuire, had died in the usual course of events. His fellow-laborers and fellow members of the beneficiary association, of which he was a shining light, held a memorial service before the body was taken to the church. The chief-speaker was one of those oratorically inclined gentlemen whose poetic license goes untagged. In the course of his remarks, he said that the deceased was a useful member of the society. A saddened and sleepy brother, who had been drowning his sorrow, woke up in time to hear the words and broke in with: "Useful? Use? of what use? He's a dead one." But to return to the book. It is nicely gotten out. The State Journal Press does good printing. The binder knew his business, and they certainly have got a good photo-engraving plant in Omaha where it was published, or in Lincoln where it was printed, for the half-tones, while they are not instructive in any sense are very excellent exhibitions of the mechanics of latter-day illustrating.

As to the vitals of the book, a passing word will suffice. In the 149 pages, everything is covered—general and special surgery from the fascinating field of brain lesions, down to thoracic-tinkering, still further down to the abdomen, that fertile pasture whereof it has been said by the specialist of a big city that "they open up the belly and go in with a milking stool, sit down and tinker around." And still on, on, on, on with an excelsior sort of a rush, the author crashes into amputations, sprains and what not, with now and then a dash into a sea of pus, and a plunge into the horrors of tetanus and "septic blood poisoning."

Then, too, there is an exposé of bacteriology, in which we are given a new classification under the heading of "Divisions of micro-organisms," as follows: (1) "micrococci, which are cells, either round or oval;" (2) "bacilli, which are rod-shaped cells," and (3) "bacteria, which may be rod-shaped; . . . . also oval."

I read the book, but I did it as a duty and some duties are so disagreeable that they have a leathery taste in one's mouth. As I have said the printing, the binding and the pictures are good. As for the book as a book, well, read that McGuire story again!

